# Long‐term safety and effectiveness of berotralstat for hereditary angioedema: The open‐label APeX‐S study

**DOI:** 10.1002/clt2.12035

**Published:** 2021-06-18

**Authors:** Henriette Farkas, Marcin Stobiecki, Jonny Peter, Tamar Kinaciyan, Marcus Maurer, Emel Aygören‐Pürsün, Sorena Kiani‐Alikhan, Adrian Wu, Avner Reshef, Anette Bygum, Olivier Fain, David Hagin, Aarnoud Huissoon, Miloš Jeseňák, Karen Lindsay, Vesna Grivcheva Panovska, Urs C. Steiner, Celia Zubrinich, Jessica M. Best, Melanie Cornpropst, Daniel Dix, Sylvia M. Dobo, Heather A. Iocca, Bhavisha Desai, Sharon C. Murray, Eniko Nagy, William P. Sheridan

**Affiliations:** ^1^ Hungarian Angioedema Center of Reference and Excellence Department of Internal Medicine and Hematology Semmelweis University Budapest Hungary; ^2^ Department of Clinical and Environmental Allergology Jagiellonian University Medical College Krakow Poland; ^3^ Allergy and Immunology Unit University of Cape Town Lung Institute Cape Town South Africa; ^4^ Division of Allergy and Clinical Immunology Department of Medicine University of Cape Town Cape Town South Africa; ^5^ Department of Dermatology Medical University of Vienna Vienna Austria; ^6^ Dermatological Allergology Department of Dermatology and Allergy Charité ‐ Universitätsmedizin Berlin Berlin Germany; ^7^ Department for Children and Adolescents University Hospital Frankfurt Goethe University Frankfurt Frankfurt Germany; ^8^ Department of Immunology Barts Health NHS Trust Royal London Hospital London UK; ^9^ Center for Allergy and Asthma Care Central Hong Kong China; ^10^ Angioderma Center Barzilai University Medical Center Ashkelon Israel; ^11^ Department of Dermatology and Allergy Centre Odense University Hospital Odense Denmark; ^12^ Department of Clinical Genetics Odense University Hospital Odense Denmark; ^13^ Clinical Institute University of Southern Denmark Odense Denmark; ^14^ Sorbonne Université Service de Médecine Interne AP‐HP, Hôpital Saint‐Antoine Paris France; ^15^ Allergy and Clinical Immunology Unit Department of Medicine Tel Aviv Sourasky Medical Center and Sackler Faculty of Medicine University of Tel Aviv Tel Aviv Israel; ^16^ Department of Immunology Birmingham Heartlands Hospital University Hospitals Birmingham UK; ^17^ National Center for Hereditary Angioedema Department of Pediatrics Department of Pulmonology and Allergology Comenius University in Bratislava Jessenius Faculty of Medicine Martin Slovakia; ^18^ Auckland DHB Clinical Immunology and Allergy Auckland New Zealand; ^19^ University Clinic of Dermatology Ss Cyril and Methodius University Skopje Macedonia; ^20^ Department of Immunology University Hospital Zurich Zurich Switzerland; ^21^ Allergy, Asthma and Clinical Immunology Alfred Health Melbourne Victoria Australia; ^22^ BioCryst Pharmaceuticals Durham North Carolina USA

**Keywords:** berotralstat, hereditary angioedema, long‐term, prophylaxis, safety

## Abstract

**Background:**

Berotralstat (BCX7353) is an oral, once‐daily inhibitor of plasma kallikrein recently approved for prevention of angioedema attacks in adults and adolescents with hereditary angioedema (HAE). The objective of this report is to summarize results from an interim analysis of an ongoing long‐term safety study of berotralstat in patients with HAE.

**Methods:**

APeX‐S is an ongoing, phase 2, open‐label study conducted in 22 countries (ClinicalTrials.gov, NCT03472040). Eligible patients with a clinical diagnosis of HAE due to C1 inhibitor deficiency (HAE‐C1‐INH) were centrally allocated to receive berotralstat 150 or 110 mg once daily. The primary objective was to determine long‐term safety and the secondary objective was to evaluate effectiveness.

**Results:**

Enrolled patients (*N* = 227) received berotralstat 150 mg (*n* = 127) or 110 mg (*n* = 100) once daily. The median (range) duration of exposure was 342 (11–540) and 307 (14–429) days for the 150‐mg and 110‐mg groups, respectively. Treatment‐emergent adverse events (TEAEs) occurred in 91% (*n* = 206) of patients. The most common TEAEs across treatment groups were upper respiratory tract infection (*n* = 91, 40%), abdominal pain (*n* = 57, 25%), headache (*n* = 40, 18%), and diarrhea (*n* = 31, 14%) and were mostly mild to moderate. Fifty percent (*n* = 113) of patients had at least one drug‐related adverse event (AE; 150 mg, *n* = 57 [45%]; 110 mg, *n* = 56 [56%]), and discontinuations due to AEs occurred in 19 (8%) patients (150 mg, *n* = 13 [10%]; 110 mg, *n* = 6 [6%]). Three (1.3%) patients experienced a drug‐related serious TEAE. Among patients who received berotralstat through 48 weeks (150 mg, *n* = 73; 110 mg, *n* = 30), median HAE attack rates were low in month 1 (150 mg, 1.0 attacks/month; 110 mg, 0.5 attacks/month) and remained low through 12 months (0 attacks/month in both dose groups). Mean HAE attack rates followed a similar trend, and no evidence for patient tolerance to berotralstat emerged. In both dose groups, angioedema quality of life scores showed clinically meaningful changes from baseline.

**Conclusions:**

In this analysis, both berotralstat doses, 150  and 110 mg once daily, were generally well tolerated. Effectiveness results support the durability and robustness of berotralstat as prophylactic therapy in patients with HAE.

**Trial registration:**

The study is registered with ClinicalTrials.gov (NCT03472040).

## 1 INTRODUCTION

Hereditary angioedema (HAE) due to C1 inhibitor (C1‐INH) deficiency (HAE‐C1‐INH) is a genetic disease characterized by recurrent attacks of subcutaneous and/or submucosal swelling.^1^, ^2^ HAE‐C1‐INH is caused by either quantitative deficiencies or dysfunctional production of C1‐INH, leading to uncontrolled plasma kallikrein activity, excessive release of bradykinin, and consequent angioedema.^3^, ^4^ HAE attacks most commonly affect the subcutaneous tissues of the extremities, torso, face, and genitals, and the submucosal tissues of the intestines, oropharynx, and larynx.^5^ The onset of attacks is generally not predictable, and attacks with laryngeal angioedema can be life‐threatening because of potential upper airway obstruction and asphyxiation.^6^, ^7^ HAE attacks can be painful and incapacitating, interfering with a patient’s ability to perform daily activities.^8^, ^9^ These attacks can lead to emergency room visits and hospitalization, often with incorrect diagnoses and ineffective treatments for misdiagnosed patients.^7^, ^10^ The variable frequency of attacks, the risk of laryngeal edema, and a familial history of fatal laryngeal attacks can lead to anxiety and depression, contributing to a significant burden of disease and reduced quality of life (QoL) for patients.^11^, ^12^, ^13^, ^14^

Intravenously (IV) or subcutaneously (SC) administered on‐demand treatments reduce the severity and duration of HAE attacks.^15^, ^16^, ^17^ Recent treatment guidelines advise that a prophylactic treatment strategy should be considered in every patient with HAE to reduce the frequency and severity of angioedema attacks and that the decision to use long‐term prophylaxis should reflect the needs of the individual patient.^17^, ^18^ Historically, prophylactic treatment options included oral attenuated androgens and antifibrinolytics. Antifibrinolytics are not efficacious,^19^ and oral attenuated androgens have numerous side effects that limit their tolerability, particularly for women and children.^20^, ^21^, ^22^ In the past 10 years, targeted therapies including IV or SC formulations of C1‐INH replacement therapy,^19^, ^23^, ^24^ an SC plasma kallikrein inhibitor (lanadelumab),^25^ and an oral plasma kallikrein inhibitor (berotralstat)^26^ have been shown to substantially reduce disease activity.^25^ Administration of parenteral therapies may require caregivers’ or healthcare professional assistance, adding a significant treatment burden, which may be difficult for some patients, particularly pediatric patients, those with a fear of needles, or those with poor peripheral venous access.^15^, ^16^, ^27^ Because HAE is a lifelong disease, there is a need for long‐term treatment options that are efficacious, well tolerated, and have a lower treatment burden.^28^

Orladeyo® (berotralstat) is an oral, once‐daily, highly selective inhibitor of plasma kallikrein recently approved for prevention of angioedema attacks in adults and adolescents with HAE.^29^ In the primary analysis of the phase 3, randomized, double‐blind, placebo‐controlled APeX‐2 trial (NCT03485911), treatment with berotralstat 150  and 110 mg was shown to significantly reduce HAE attack rates compared with placebo through 24 weeks (150 mg: 1.31 attacks/month, *p* < 0.001; 110 mg: 1.65 attacks/month, *p* = 0.024; placebo: 2.35 attacks/month).^26^ These results were further supported by the primary analysis (24 weeks) of the phase 3, randomized, double‐blind, placebo‐controlled APeX‐J trial (NCT03873116) demonstrating that treatment with 150 mg of berotralstat significantly reduced HAE attacks relative to placebo (1.11 vs. 2.18 attacks/month, *p* = 0.003) in patients with HAE in Japan.^30^

Here, we report results of a planned interim analysis of the ongoing, global, open‐label APeX‐S study, which was designed to evaluate the long‐term safety and effectiveness of oral once‐daily berotralstat 150  and 110 mg for the prevention of angioedema attacks in patients with HAE.

## 2 METHODS

### 2.1 Study design

APeX‐S is an ongoing, global, open‐label, nonrandomized study in which enrolled patients were centrally allocated to receive 150 or 110 mg of open‐label berotralstat. The current analysis includes patients at 49 sites in 22 countries (Table S1). The study was initially designed to evaluate the safety and effectiveness of a single dose level of berotralstat (150 mg once daily) over 48 weeks of treatment but was amended to add a second dose level (110 mg once daily) based on the inclusion of both doses (150  and 110 mg) in the pivotal phase 3 APeX‐2 randomized control trial. The current preplanned data analysis occurred when approximately 100 patients treated with 150 mg of berotralstat from a combination of this study and the APeX‐2 trial completed 48 weeks of treatment. The APeX‐S study remains ongoing. This report summarizes results for all enrolled patients from February 27, 2018 (first patient enrolled) through August 20, 2019 (ClinicalTrials.gov, NCT03472040).

This study was designed, performed, and monitored in accordance with Good Clinical Practice guidelines according to the International Council on Harmonisation and in compliance with the Declaration of Helsinki. The protocol, amendments, informed consent forms, and other relevant study documentation were approved by institutional review boards and independent ethics committees before implementation in accordance with regulatory requirements.

### 2.2 Patients

Patients with HAE were eligible for the study in all participating countries if aged ≥18 years and if aged ≥12 to 17 years in certain countries (where allowed). Enrolled patients must have either participated in a previous berotralstat study or be expected to benefit from berotralstat treatment in the opinion of the investigator and have fulfilled all other inclusion criteria. For patients naive to berotralstat treatment, diagnosis of HAE‐C1‐INH was confirmed by C1‐INH functional level (<50% by chromogenic assay) and complement 4 (C4) level below the lower limit of normal (LLN) as assessed during the screening period. Patients with a C1‐INH functional level between 50% and the assay LLN (74%) or a C4 value above the LLN could qualify via alternative protocol‐specified criteria (Supporting Information: Methods). Patients who met eligibility criteria for and participated in a prior berotralstat study were not required to requalify under this diagnostic criterion at the time of enrollment in APeX‐S. Eligible patients were required to have access to at least one appropriate on‐demand rescue medication to treat HAE attacks. Sites requested a treatment allocation for eligible patients in the interactive voice/web response system (IxRS).

### 2.3 Procedures

Safety was evaluated by assessment of treatment‐emergent adverse events (TEAEs) and laboratory analyses. Definitions and assessment of severity of adverse events (AEs) were based on criteria listed in the 2007 Division of Microbiology and Infectious Diseases Adult Toxicity Table.^31^ The relationship of an AE to study drug was assessed by the investigator or medically qualified designee. TEAEs of rash were required to be assessed and reported promptly for review by the medical monitor. Patients were required to discontinue berotralstat if they had liver enzyme elevations that exceeded protocol‐specified levels or met other study‐specified stopping rules.

At the baseline visit, patients were informed of the importance of diary completion and appropriate recording of events (i.e., reporting attacks in the diary and other symptoms to the investigator). Data on angioedema attacks, including the location of symptoms, severity, and treatment(s) administered, were recorded by patients daily using a paper diary. Attacks were adjudicated (confirmed or rejected for inclusion in the effectiveness analysis) using a programmed algorithm according to predefined criteria created in collaboration with HAE‐treating physicians and defined in the statistical analysis plan. A patient‐reported attack must have met the following criteria to be confirmed: (1) the attack must have included ≥1 symptom of swelling, (2) there must not have been an alternative explanation for the swelling (e.g., allergic reaction), (3) any attack that began within 24 h of the end of the previous attack was combined with the previous attack, and (4) if untreated, the attack must have had a duration of >24 h. Treatment of attacks followed the patients’ normal treatment plan. Disease‐specific QoL impairment was measured with the Angioedema QoL Questionnaire (AE‐QoL).^32^ This validated, patient‐reported outcome measure assesses QoL in 4 dimensions with a total of 17 items; a lower total score indicates improved QoL (scale: 0–100 points; the minimum clinically important difference [MCID] is a change of 6 points in total score).^32^, ^33^ Patients’ satisfaction with their medication was assessed with the Treatment Satisfaction Questionnaire for Medication (TSQM).^34^ The TSQM consists of 14 items. Scores range from 0 to 100, with higher scores indicating higher satisfaction.^34^ Questionnaires were not administered in countries where validated translations did not exist (AE‐QoL: Hong Kong, Serbia, and South Korea; TSQM: Hong Kong).

Patients were instructed to take berotralstat capsules orally, once daily at the same time each day with their largest meal or within 30 min of that meal. Percent study drug compliance was based upon returned capsule count and computed as the number of capsules taken divided by the expected number of capsules × 100.

### 2.4 Objectives and outcome measures

The primary objective of the study was to evaluate the long‐term safety and tolerability of daily dosing of oral berotralstat in patients with HAE. Safety endpoints included the proportion of patients with TEAEs, grade 3 (severe) or 4 (life‐threatening) TEAEs, treatment‐emergent serious adverse events (TESAEs), discontinuations due to TEAEs, drug‐related TEAEs consistent with a drug rash, and treatment‐emergent grade 3 or 4 laboratory abnormalities. The secondary objectives of the study were to assess effectiveness, QoL, and patient satisfaction during long‐term administration of berotralstat. Effectiveness endpoints included the number and rate of HAE attacks, the durability of response (attack‐rate trend over time), the proportion of days with angioedema symptoms, the number of attacks requiring treatment, discontinuations due to lack of efficacy, severity of attacks, and patient‐reported outcomes (AE‐QoL, TSQM).

### 2.5 Statistical analysis

All safety and effectiveness analyses were conducted for the safety population (all patients receiving ≥1 dose of study drug). Demographics, disease characteristics, compliance with study treatment, and safety endpoints were summarized by treatment group. AEs were assessed and recorded from the time the informed consent form was signed through the end‐of‐study visit. TEAEs were defined as AEs that occurred on or after the initiation of study drug and up to 30 days after the last dose of study drug. Unless otherwise specified, TEAEs are reported throughout this manuscript. In this study, HAE attacks and their associated symptoms were not defined as TEAEs unless they met the criteria for a serious adverse event (SAE). Patient incidence of AEs and rate per 100 person‐years of exposure (PYE) were tabulated. Clinical laboratory results were summarized using descriptive statistics. Subgroup analyses of selected safety endpoints were conducted based on latency of prior androgen use (i.e., time between discontinuing the androgen and starting berotralstat) and duration of prior androgen use. An independent data monitoring committee provided safety monitoring at prespecified intervals with additional consultation or review as needed.

For effectiveness analyses, patient‐reported and programmatically adjudicated HAE attacks were analyzed by treatment group using descriptive statistics. All subsequent discussions of HAE attacks in this manuscript refer to programmatically adjudicated HAE attacks unless otherwise specified. Some patients were discontinuing other prophylactic therapies prior to entry and other patients were entering from a prior berotralstat study; therefore, no baseline attack rate was collected. For the safety population, attack rates were computed by month (1 month = 28 days) as well as for the entire dosing period. Ad hoc analyses of attack rates were performed for patients who completed 48 weeks of berotralstat treatment. The proportion of days with angioedema symptoms were summarized using descriptive statistics by treatment group and presented by month and for the entire dosing period. Discontinuations due to perceived lack of efficacy were summarized. The patient‐reported attack severity for adjudicated attacks as well as the percentage of adjudicated attacks treated with SOC medication were summarized descriptively. Changes from baseline in AE‐QoL total scores and TSQM global satisfaction scores were calculated and summarized.

## 3 RESULTS

### 3.1 Study population

**FIGURE 1:**
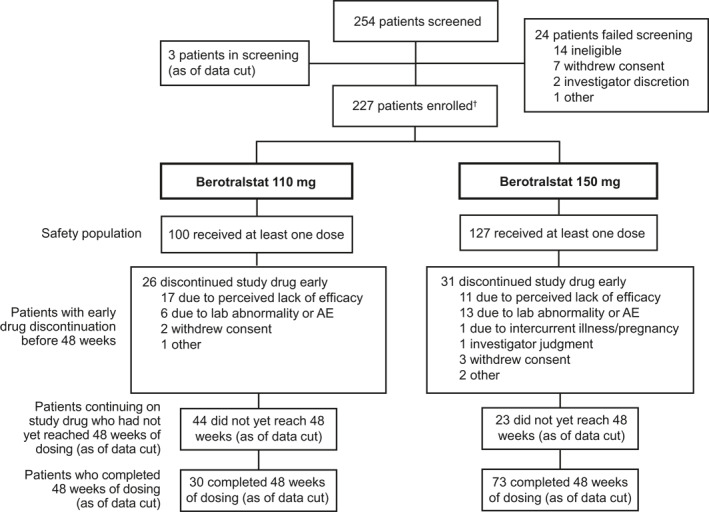
Patient disposition. AE, adverse event. ^†^Patients were centrally allocated to one of two treatment groups. At the request of the Ministry of Food and Drug Safety, patients in South Korea were randomized 1:1 between the treatment groups

Of 254 screened patients, 227 were centrally allocated to study drug and received ≥1 dose of study treatment (150 mg, *n* = 127; 110 mg, *n* = 100). The majority of enrolled patients had not participated in a previous berotralstat trial (*n* = 151, 67%). At the cutoff date for this analysis, 73 patients receiving 150 mg and 30 patients receiving 110 mg had completed 48 weeks of study drug dosing (Figure 1). Overall, 57 (25%) patients discontinued study drug before 48 weeks, and 67 (30%) had not yet reached 48 weeks of dosing (23 receiving 150 mg and 44 receiving 110 mg). The most common reasons for discontinuation were perceived lack of efficacy (*n* = 28, 12%) and laboratory abnormalities or AEs (*n* = 19, 8%; Figure 1). The mean (standard deviation [SD]) compliance with study drug dosing was 95% (9.0).

Baseline demographics were generally well balanced between treatment groups except for age, with patients receiving berotralstat 150 mg being older by approximately 5 years on average (Table 1). Ten adolescents (aged ≥12–17 years) were enrolled. Overall, 139 (61%) of patients were female. Most patients (*n* = 183, 81%) had used at least one past prophylactic treatment, the most common being attenuated androgens (*n* = 142, 63%). With berotralstat 150 mg, 18 (14%) patients discontinued attenuated androgens <2 weeks before the first dose of berotralstat compared with 22 (22%) patients receiving berotralstat 110 mg.

**TABLE 1:** Baseline characteristics

Patient characteristics	Berotralstat 110 mg (*n* = 100)	Berotralstat 150 mg (*n* = 127)	Total (*N* = 227)
Age, mean (SD), y	37.6 (14.0)	42.5 (13.4)	40.3 (13.9)
Age, *n* (%)			
12–17 years	5 (5)	5 (4)	10 (4)
18–64 years	93 (93)	119 (94)	212 (93)
≥65 years	2 (2)	3 (2)	5 (2)
Female, *n* (%)	62 (62)	77 (61)	139 (61)
Weight, mean (SD), kg	75.7 (19.1)	78.7 (18.9)	77.4 (19.0)
Race, *n* (%)			
Asian	12 (12)	10 (8)	22 (10)
White	82 (82)	110 (87)	192 (85)
Other	6 (6)	7 (6)	13 (6)
Any prior prophylactic treatment for HAE, *n* (%)	81 (81)	102 (80)	183 (81)
Any prior androgen use	61 (61)	81 (64)	142 (63)
Any prior C1‐INH use^a^	22 (22)	32 (25)	54 (24)
Time since discontinuation of attenuated androgens			
Never used	31 (31)	43 (34)	74 (33)
<2 weeks	22 (22)	18 (14)	40 (18)
2 weeks to <1 month	4 (4)	5 (4)	9 (4)
1 month to <2 months	8 (8)	5 (4)	13 (6)
≥2 months	35 (35)	56 (44)	91 (40)

Abbreviations: C1‐INH, C1 inhibitor; HAE, hereditary angioedema; SD, standard deviation.

^a^ C1‐INH includes plasma‐derived and recombinant human C1‐INH and fresh frozen plasma.

### 3.2 Safety

#### 3.2.1 Treatment‐emergent AEs

The median (range) duration of exposure was 342 (11–540) days for berotralstat 150 mg and 307 (14–429) days for 110 mg. Overall, there were 107 PYE for berotralstat 150 mg and 69 PYE for 110 mg. The safety profiles were similar between doses and therefore, safety is presented across both doses. TEAEs were reported in 91% of patients during the treatment period. The most frequently reported AEs were upper respiratory tract infection (*n* = 91, 40%), abdominal pain (*n* = 57, 25%), headache (*n* = 40, 18%), and diarrhea (*n* = 31, 14%) (Table 2). A similar trend in frequent AEs was observed when the data were reviewed based on AE frequency per 100 PYE (Table S2). Fifty percent of patients had at least 1 drug‐related AE (150 mg, *n* = 57 [45%]; 110 mg, *n* = 56 [56%]). The most common drug‐related events across both treatment groups were abdominal pain (*n* = 22, 9.7%), diarrhea (*n* = 16, 7.0%), and nausea (*n* = 14, 6.2%). AEs were largely mild to moderate in severity. Thirty‐four (15%) patients experienced a grade 3 or 4 TEAE, with HAE attack (*n* = 8, 3.5%), increased alanine aminotransferase (ALT; *n* = 6, 2.6%), increased hepatic enzyme (*n* = 2, 0.9%), abnormal liver function test (LFT; *n* = 2, 0.9%), and pneumonia (*n* = 2, 0.9%) as the most frequently reported. Six percent of patients had drug‐related grade 3 or 4 TEAEs. Thirty (13%) patients experienced a TESAE (150 mg, *n* = 12 [9%]; 110 mg, *n* = 18 [18%]), with half related to HAE attacks (12 patients) and medical observation (three patients). This may be attributed to hospitalization being a common practice for HAE attacks in some of the regions, leading to classification of these events as TESAEs. Drug‐related SAEs were reported in three (1.3%) patients (150 mg, *n* = 1 [0.8%]; 110 mg, *n* = 2 [2.0%]); one patient on berotralstat 150 mg had abnormal LFT (began on day 18 and resolved on day 56; 37 days after withdrawal from study), one patient on berotralstat 110 mg had increased hepatic enzyme (began on day 18 and resolved on day 118) and gastroenteritis (began on day 11 and resolved on day 23; 5 days after withdrawal from study), and one patient on berotralstat 110 mg had abdominal pain (began on day 6 and decreased to grade 1 on day 36; 29 days after treatment interruption without recurrence of symptoms, and the event was resolved).

**TABLE 2:** Overall summary of adverse events

Safety outcomes, *n* (%)	Berotralstat 110 mg (*n* = 100)	Berotralstat 150 mg (*n* = 127)	Total (*N* = 227)
Any AE	91 (91)	115 (91)	206 (91)
Most common AEs with medical concepts (>10% of patients in any treatment group)^a^			
Upper respiratory tract infection	34 (34)	57 (45)	91 (40)
Abdominal pain	31 (31)	26 (21)	57 (25)
Headache	21 (21)	19 (15)	40 (18)
Diarrhea	13 (13)	18 (14)	31 (14)
Drug‐related AE	56 (56)	57 (45)	113 (50)
Grade 3 or 4 AE	14 (14)	20 (16)	34 (15)
Drug‐related grade 3 or 4 AE^b^	6 (6)	8 (6)	14 (6)
Investigator‐identified drug‐related rash (EOSI)^c^	2 (2)	0 (0)	2 (1)
SAE	18 (18)	12 (9)	30 (13)
Drug‐related SAE	2 (2)	1 (1)	3 (1)

Abbreviations: AE, adverse event; EOSI, event of special interest; SAE, serious adverse event.

^a^ Adverse events are coded using MedDRA version 19.1. The terms “abdominal pain,” “diarrhea,” and “upper respiratory tract infection” are medical concepts containing multiple preferred terms. “Abdominal pain” contains the preferred terms “abdominal pain,” “abdominal discomfort,” “abdominal pain upper,” “abdominal pain lower,” and “epigastric discomfort.” “diarrhea” contains the preferred terms, “diarrhea” and “feces soft.” “upper respiratory tract infection” contains the preferred terms “nasopharyngitis,” “upper respiratory tract infection,” “viral upper respiratory tract infection,” “respiratory tract infection,” and “respiratory tract infection viral.”

^b^ Berotralstat 110 mg (*n* = number of patients): Alanine aminotransferase increased (*n* = 3), hepatic enzyme increased and gastroenteritis (*n* = 1), liver function test abnormal (*n* = 1), abdominal pain (*n* = 1). Berotralstat 150 mg (*n* = number of patients): Alanine aminotransferase increased (*n* = 2), four events of increased alanine aminotransferase (*n* = 1), hepatic enzyme increased (*n* = 1), liver function test abnormal (*n* = 1), transaminases increased (*n* = 1), arthralgia and myalgia (*n* = 1), dizziness and headache (*n* = 1). Patients may have experienced the same coded event more than once.

^c^ Diffuse maculopapular rash assessed as at least possibly related to berotralstat treatment.

Overall, 10% (*n* = 13) and 6% (*n* = 6) of patients receiving berotralstat 150  and 110 mg, respectively, discontinued study drug due to AEs occurring primarily within the first month of treatment (Table 3). The AEs or laboratory abnormalities leading to discontinuation in >1 patient were liver transaminase elevations (eight patients), upper abdominal pain (three patients), and vomiting (two patients).

**TABLE 3:** Overall summary of adverse events leading to discontinuation of study drug

Safety outcomes, *n* (%)	Berotralstat	Berotralstat	Total (*N* = 227)
	110 mg (*n* = 100)	150 mg (*n* = 127)	
AEs leading to discontinuation of study drug^a^	6 (6)	13 (10)	19 (8)
AEs or laboratory abnormalities leading to discontinuation of study drug (>1 patient in any treatment group)			
Liver‐related abnormalities	3 (3)	5 (4)	8 (4)
Upper abdominal pain	2 (2)	1 (1)	3 (1)
Vomiting	0 (0)	2 (2)	2 (1)

Abbreviation: AE, adverse event.

^a^ Berotralstat 110 mg (*n* = number of patients): Alanine aminotransferase and aspartate aminotransferase increased (*n* = 1), hepatic enzyme increased and drug eruption and gastroenteritis (*n* = 1), liver function test abnormal (*n* = 1), abdominal pain upper (*n* = 2), headache (*n* = 1). Berotralstat 150 mg (*n* = number of patients): Alanine aminotransferase increased (*n* = 1), alanine aminotransferase and aspartate aminotransferase increased (*n* = 1), hepatic enzyme increased (*n* = 1), liver function test abnormal (*n* = 1), abdominal pain upper (*n* = 1), vomiting (*n* = 1), diarrhea and alanine aminotransferase increased (*n* = 1), vomiting and nausea (*n* = 1), dermatitis psoriasiform and rash (*n* = 1), anemia (*n* = 1), arthralgia, myalgia, and dizziness (*n* = 1), acute myelomonocytic leukemia (*n* = 1), depression and suicide attempt (*n* = 1). Patients may have experienced the same coded event more than once.

Seven (70%) of the 10 adolescent patients experienced a TEAE (150 mg, *n* = 5 [100%]; 110 mg, *n* = 2 [40%]). The most frequently reported events in adolescent patients were headache (*n* = 3, 30%), nasopharyngitis (*n* = 2, 20%), and sinusitis (*n* = 2, 20%). Three experienced a drug‐related AE (abdominal pain, *n* = 1; abdominal pain upper, *n* = 1; headache and two events of dizziness, *n* = 1). Two adolescent patients experienced at least one grade 3 or 4 TEAE: one patient experienced facial paralysis not considered related to berotralstat, and the other experienced headache and dizziness that were both considered possibly related to berotralstat (all were resolved). One adolescent patient in the 110‐mg group discontinued study drug due to grade 2 upper abdominal pain.

Eight (3.5%) patients had a rash assessed by the investigator as related to study drug; in two (0.9%) patients, the event was consistent with delayed drug hypersensitivity reaction presenting as a benign generalized maculopapular rash, which had been reported in previous studies. Both events were grade 1 and resolved, with one patient remaining on berotralstat.

### 3.3 Laboratory abnormalities

The most common clinically significant treatment‐emergent laboratory abnormality was elevated ALT. Thirteen (6%) patients had a treatment‐emergent grade 3 or 4 ALT abnormality (150 mg, *n* = 6; 110 mg, *n* = 7), most commonly within the first month after berotralstat exposure. All 13 had previously used androgens. All but one of the 13 patients had stopped androgens 7 to 9 days before the first dose of berotralstat; one patient stopped androgens <2 months from the first dose of berotralstat. Patients who discontinued androgens <2 weeks before starting berotralstat were more likely to experience a TEAE of ALT elevation (25%) compared with those who had never used androgens (1%) or those who discontinued androgens ≥2 weeks prior (3%). ALT elevations were generally transient with no evidence of jaundice or synthetic dysfunction and resolved or improved during the observation period; six patients discontinued berotralstat, five patients continued berotralstat, and two patients subsequently discontinued because of an unrelated perceived lack of efficacy.

### 3.4 Effectiveness

#### 3.4.1 HAE attack rates

**FIGURE 2:**
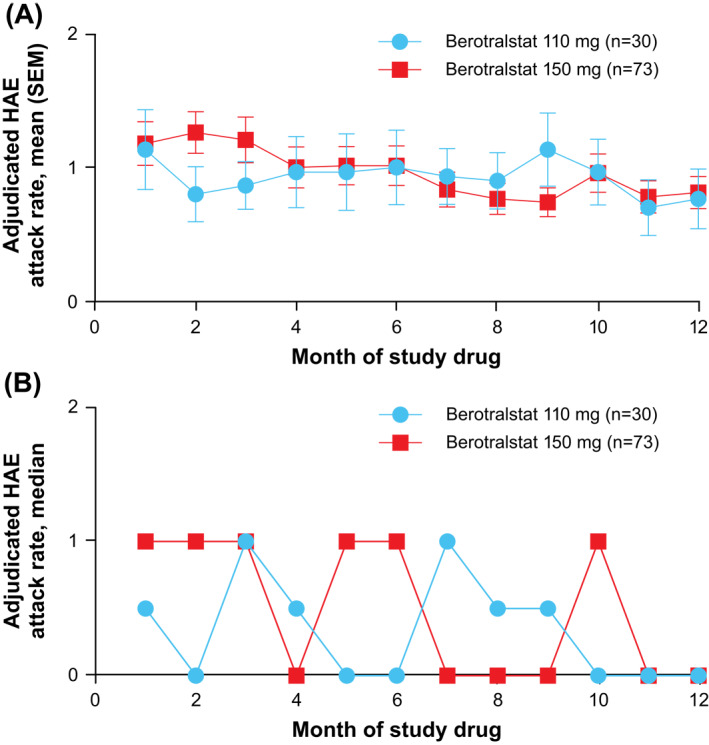
Mean (A) and median (B) adjudicated monthly HAE attack rate for patients completing 48 weeks of dosing. HAE, hereditary angioedema; SEM, standard error of the mean

Among patients receiving berotralstat through week 48, mean (SD) attack rates at month 1 were 1.2 (1.4) attacks/month and 1.1 (1.6) attacks/month with berotralstat 150  and 110 mg, respectively. The mean (SD) rate of HAE attacks generally declined through month 12, with 0.8 attacks/month reported in month 12 in both treatment groups (Figure 2A). Median attack rates followed a similar trend. Patients receiving 150  and 110 mg of berotralstat had median (range) attack rates of 1.0 (0.0–7.0) and 0.5 (0.0–7.0) attacks/month at month 1, respectively (Figure 2B). Both groups had a median (range) attack rate of 0.0 (0.0–4.0) attacks/month at month 12.

In an analysis of adjudicated HAE attack rates by age group, adolescent patients aged 12 to 17 years (*n* = 10) had mean (SD) attack rates over the entire treatment period of 0.77 (0.96) attacks/month and 0.78 (1.02) attacks/month with 150 mg (*n* = 5) and 110 mg (*n* = 5), respectively, while on berotralstat treatment.

Throughout the study, 76% of attacks were reported by patients as negligible, mild, or moderate in severity. Of the on‐study attacks, 82% were treated with SOC medication. Among treated attacks, the SOC medications used for the treatment of attacks were plasma‐derived C1‐INH (46%), icatibant (38%), recombinant C1‐INH (4%), and fresh frozen plasma (1%).

### 3.5 Days with angioedema symptoms

In the entire study population, the median (range) proportion of days with angioedema symptoms was low across the treatment period (150 mg, 0.07 [0–0.7], equivalent to 23.5 days during a 48‐week period; 110 mg, 0.08 [0–0.7], equivalent to 26.9 days during a 48‐week period), where the proportion of days is based on the number of days that the patient was on treatment.

### 3.6 QoL and treatment satisfaction scores

**FIGURE 3:**
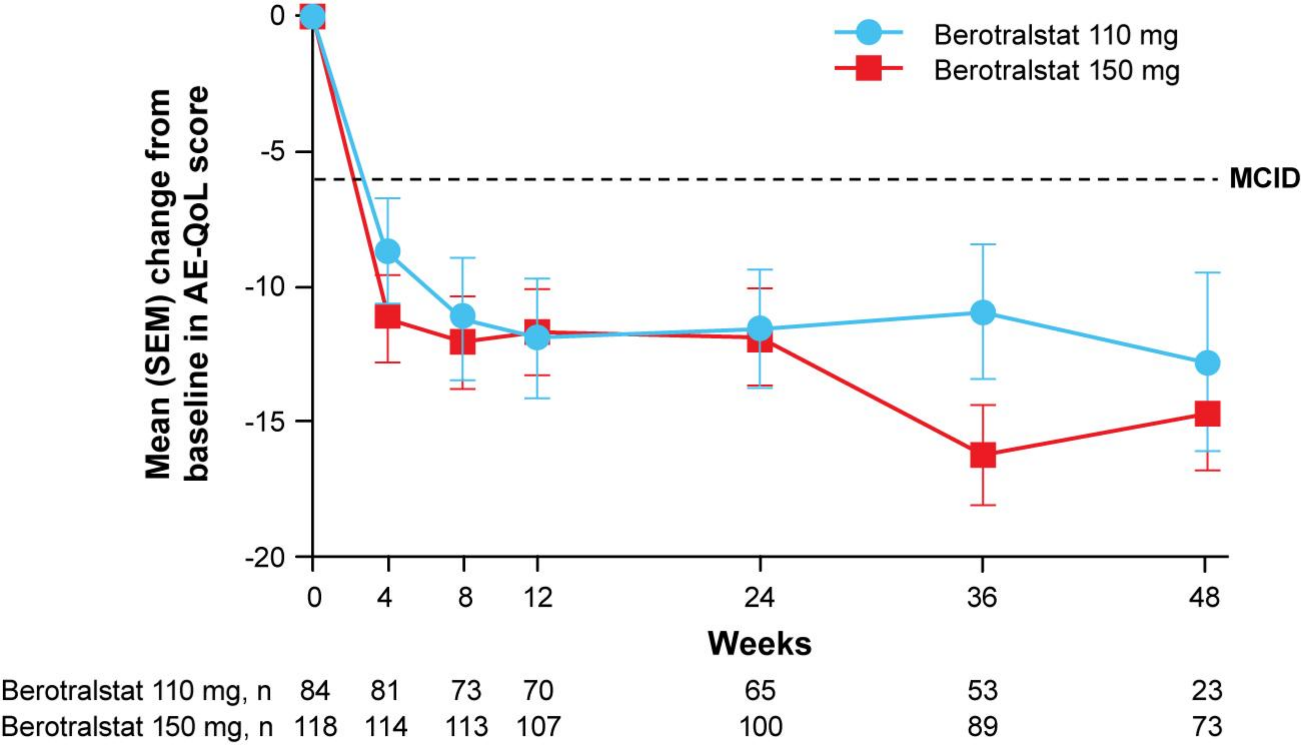
Plot of mean (SEM) change from baseline in AE‐QoL scores over time. AE‐QoL, Angioedema Quality of Life Questionnaire; MCID, minimum clinically important difference; SEM, standard error of the mean

Clinically meaningful mean (SD) changes in AE‐QoL total scores were observed in both dose groups from baseline (150 mg, 37.8 [17.9]; 110 mg, 42.2 [21.2]) to week 48 (150 mg, 24.7 [17.8]; 110 mg, 24.9 [16.4]), demonstrating improved QoL. Improvements exceeding the MCID of 6 points were observed as early as week 4 (first postbaseline assessment) for both dose groups (Figure 3). At week 48, 62% (45/73) of patients receiving berotralstat 150 mg and 70% (16/23) of patients receiving 110 mg had change from baseline that was greater than or equal to the MCID (≥6 points) in AE‐QoL scores. TSQM scores improved from baseline to week 48 in all domains, with similar changes from baseline in both the dose groups.

## 4 DISCUSSION

The ongoing, open‐label, phase 2, APeX‐S study is a large, geographically diverse, long‐term study to assess the safety and effectiveness of berotralstat in patients with HAE‐C1‐INH. This planned data analysis demonstrated that long‐term treatment with once‐daily oral berotralstat 150 or 110 mg was well tolerated.

In this study, the most common drug‐related AEs across both treatment groups were abdominal pain, diarrhea, and nausea. These findings were consistent with observations from the phase 3, randomized, double‐blind, placebo‐controlled APeX‐2 study, in which the most common TEAEs that occurred with berotralstat compared with placebo were abdominal pain, vomiting, diarrhea, and back pain.^26^ Drug‐related SAEs were infrequent and rates of study discontinuation due to AEs were low. The most common grade 3 or 4 laboratory abnormality was ALT elevation. Notably, almost all patients reporting a grade 3 or 4 ALT increase had discontinued androgens <2 weeks before starting berotralstat. Literature reports indicate that prolonged androgen use and higher doses may be associated with an increased risk of AEs, including abnormal LFT results.^22^, ^35^, ^36^, ^37^ The observed safety profile of berotralstat from this study was generally consistent with the pivotal phase 3, placebo‐controlled APeX‐2 study, with no new safety signals observed.^26^

Among patients who received berotralstat for 48 weeks, low attack rates were observed within the first 4 weeks of treatment and were maintained or improved through week 48. Berotralstat was previously shown to reduce HAE attack rates over 24 weeks in the pivotal phase 3, randomized, double‐blind, placebo‐controlled APeX‐2 study.^26^ Attack rates did not increase over the course of the study, indicating that patients did not exhibit tolerance to berotralstat treatment. Mean (SD) attack rates were 1.2 (1.4) attacks/month in month 1, 1.0 (1.3) attacks/month in month 6, and 0.8 (1.0) attacks/month in month 12 for patients completing 48 weeks of treatment with 150 mg of berotralstat, and 1.1 (1.6) attacks/month in month 1, 1.0 (1.5) attacks/month in month 6, and 0.8 (1.2) attacks/month in month 12 for patients completing 48 weeks of treatment with 110 mg of berotralstat. Improvements in AE‐QoL and patient satisfaction with medication also support the durability of response in reducing HAE attack rates with berotralstat.

A limitation of this study is that patients were centrally allocated to treatment rather than randomized, introducing imbalances between treatment groups across different regions with varying local patterns of care, such as differences in previous prophylactic therapy. Another limitation is that baseline attack rates were not calculated for this study because the primary objective of this study was to assess safety; patients were allowed to enroll directly from a previous berotralstat study, while treatment‐naive patients were not required to complete a prospective run‐in period to measure baseline attack rate. Lastly, this study was noncomparative; efficacy was compared with placebo in the APeX‐2 and APeX‐J trials.^26^, ^30^

HAE is a lifelong condition and is often treated with long‐term prophylactic therapy. While other currently available prophylactic therapies for HAE, including IV and SC formulations of plasma‐derived C1‐INH^23^, ^24^ and SC lanadelumab, have been shown to be safe and effective in reducing HAE attack rates, they require preparation time and self‐infusion, which may be burdensome for patients.^25^ Berotralstat has the advantage of being an oral therapy with a simple daily dosing regimen and a lower treatment burden.

The current results of the APeX‐S study provide important data to support the safety and tolerability of berotralstat as the first targeted oral therapy for long‐term prophylaxis. To support the pivotal APeX‐2 trial, the larger study population and wide geographic distribution of patients in the APeX‐S trial allows for greater generalizability of results.^26^ This study remains ongoing to further assess long‐term safety and effectiveness of berotralstat 150 mg beyond 48 weeks of therapy.

## CONFLICT OF INTEREST

Henriette Farkas has received research grants from CSL Behring, Shire/Takeda, and Pharming; served as an advisor for CSL Behring, Shire/Takeda, Pharming, BioCryst Pharmaceuticals, and KalVista; and has participated in clinical trials/registries for BioCryst Pharmaceuticals, CSL Behring, Pharming, KalVista, and Shire/Takeda. Marcin Stobiecki reports personal fees from BioCryst during the conduct of the study; and personal fees from BioCryst, KalVista, Pharming, and Takeda (Shire) outside the submitted work. Tamar Kinaciyan reports other support from BioCryst during the conduct of the study; personal fees and grants from Shire/Takeda; and other support from CSL Behring and KalVista outside the submitted work. Marcus Maurer reports grants and personal fees from BioCryst during the conduct of the study; grants and personal fees from CSL Behring, KalVista, Shire/Takeda, and Moxie outside the submitted work; grants from Pharming; and personal fees from Pharvaris outside the submitted work. Emel Aygören‐Pürsün served as speaker for and/or advisor for and/or received grants from Adverum, BioCryst, CSL Behring, KalVista, Pharming, Pharvaris, Shire, and Takeda. Sorena Kiani‐Alikhan reports other support from BioCryst during the conduct of the study; and other support from BioCryst, Takeda, and KalVista outside the submitted work. Adrian Wu reports grants from BioCryst during the conduct of the study. Avner Reshef reports grants from BioCryst during the conduct of the study and received research grants from and served as an advisor for CSL Behring, Shire/Takeda, and Pharming. David Hagin reports other support from BioCryst during the conduct of the study. Anette Bygum has been involved in educational activities and research with the following companies: BioCryst, CSL Behring, Shire, and Takeda. Olivier Fain reports grants from BioCryst during the conduct of the study. Aarnoud Huissoon reports nonfinancial support from CSL, nonfinancial support from Biotest, and personal fees from Octapharma outside the submitted work. Miloš Jeseňák has received consultancy/speaker honoraria from CSL Behring, Shire, Sobi, and Takeda, and has served as a principal investigator for clinical trials sponsored by BioCryst, Takeda, and Pharming. Celia Zubrinich has participated in a clinical trial for BioCryst Pharmaceuticals. Karen Lindsay has received educational and research grants from Shire/Takeda. Jessica M. Best, Melanie Cornpropst, Sylvia M. Dobo, Heather A. Iocca, Bhavisha Desai, Sharon C. Murray, and William P. Sheridan are employees of BioCryst. Daniel Dix reports past employment with BioCryst. Jonny Peter, Vesna Grivcheva Panovska, and Urs C. Steiner have nothing to disclose.

## AUTHORS CONTRIBUTIONS

Henriette Farkas, Melanie Cornpropst, and Sylvia M. Dobo contributed to the conception of the study. Henriette Farkas, Emel Aygören‐Pürsün, Melanie Cornpropst, Sylvia M. Dobo, Sharon C. Murray, Eniko Nagy, and William P. Sheridan participated in the design of the study. Henriette Farkas, Marcin Stobiecki, Jonny Peter, Tamar Kinaciyan, Marcus Maurer, Emel Aygören‐Pürsün, Sorena Kiani‐Alikhan, Adrian Wu, Avner Reshef, Anette Bygum, Olivier Fain, David Hagin, Aarnoud Huissoon, Miloš Jeseňák, Karen Lindsay, Vesna Grivcheva Panovska, Urs C. Steiner, Celia Zubrinich, Daniel Dix, Sylvia M. Dobo, Heather A. Iocca, Sharon C. Murray, and Eniko Nagy were involved with acquisition of data. Jessica M. Best, Melanie Cornpropst, Daniel Dix, Sylvia M. Dobo, Heather A. Iocca, Sharon C. Murray, Eniko Nagy, and William P. Sheridan contributed to analysis of data. Henriette Farkas, Marcin Stobiecki, Jonny Peter, Tamar Kinaciyan, Marcus Maurer, Emel Aygören‐Pürsün, Sorena Kiani‐Alikhan, Adrian Wu, Avner Reshef, Jessica M. Best, Melanie Cornpropst, Sylvia M. Dobo, Heather A. Iocca, Bhavisha Desai, Sharon C. Murray, Eniko Nagy, and William P. Sheridan participated in interpretation of data. All authors contributed to the drafting, review, and revision of the manuscript and approved the final draft.

## Supporting information

Supplementary Material

## References

[1] Zuraw BL . Hereditary angioedema. N Engl J Med. 2008;359(10):1027‐1036. 18768946 10.1056/NEJMcp0803977

[2] Busse PJ , Christiansen SC . Hereditary angioedema. N Engl J Med. 2020;382(12):1136‐1148. 32187470 10.1056/NEJMra1808012

[3] Zuraw BL , Christiansen SC . HAE pathophysiology and underlying mechanisms. Clin Rev Allergy Immunol. 2016;51(2):216‐229. 27459852 10.1007/s12016-016-8561-8

[4] Kaplan AP , Joseph K . The bradykinin‐forming cascade and its role in hereditary angioedema. Ann Allergy Asthma Immunol. 2010;104(3):193‐204. 20377108 10.1016/j.anai.2010.01.007

[5] Bork K , Meng G , Staubach P , Hardt J . Hereditary angioedema: new findings concerning symptoms, affected organs, and course. Am J Med. 2006;119(3):267‐274. 16490473 10.1016/j.amjmed.2005.09.064

[6] Bork K , Hardt J , Witzke G . Fatal laryngeal attacks and mortality in hereditary angioedema due to C1‐INH deficiency. J Allergy Clin Immunol. 2012;130(3):692‐697. 22841766 10.1016/j.jaci.2012.05.055

[7] Bernstein JA . Severity of hereditary angioedema, prevalence, and diagnostic considerations. Am J Manag Care. 2018;24(14 Suppl):S292‐S298. 30132643

[8] Lumry WR , Castaldo AJ , Vernon MK , Blaustein MB , Wilson DA , Horn PT . The humanistic burden of hereditary angioedema: impact on health‐related quality of life, productivity, and depression. Allergy Asthma Proc. 2010;31(5):407‐414. 20929608 10.2500/aap.2010.31.3394

[9] Banerji A. The burden of illness in patients with hereditary angioedema. Ann Allergy Asthma Immunol. 2013;111(5):329‐336. 24125136 10.1016/j.anai.2013.08.019

[10] Zanichelli A , Longhurst HJ , Maurer M , et al. Misdiagnosis trends in patients with hereditary angioedema from the real‐world clinical setting. Ann Allergy Asthma Immunol. 2016;117(4):394‐398. 27742086 10.1016/j.anai.2016.08.014

[11] Caballero T , Aygoren‐Pursun E , Bygum A , et al. The humanistic burden of hereditary angioedema: results from the Burden of Illness Study in Europe. Allergy Asthma Proc. 2014;35(1):47‐53. 24268449 10.2500/aap.2013.34.3685

[12] Bygum A . Hereditary angioedema ‐ consequences of a new treatment paradigm in Denmark. Acta Derm Venereol. 2014;94(4):436‐441. 24202369 10.2340/00015555-1743

[13] Aygoren‐Pursun E , Bygum A , Beusterien K , et al. Estimation of EuroQol 5‐Dimensions health status utility values in hereditary angioedema. Patient Prefer Adherence. 2016;10:1699‐1707. 27660419 10.2147/PPA.S100383 PMC5019462

[14] Fouche AS , Saunders EF , Craig T . Depression and anxiety in patients with hereditary angioedema. Ann Allergy Asthma Immunol. 2014;112(4):371‐375. 24428960 10.1016/j.anai.2013.05.028 PMC4211935

[15] Tuong LA , Olivieri K , Craig TJ . Barriers to self‐administered therapy for hereditary angioedema. Allergy Asthma Proc. 2014;35(3):250‐254. 24801468 10.2500/aap.2014.35.3753

[16] Riedl MA , Banerji A , Busse PJ , et al. Patient satisfaction and experience with intravenously administered C1‐inhibitor concentrates in the United States. Ann Allergy Asthma Immunol. 2017;119(1):59‐64. 28668241 10.1016/j.anai.2017.05.017

[17] Maurer M , Magerl M , Ansotegui I , et al. The international WAO/EAACI guideline for the management of hereditary angioedema – the 2017 revision and update. World Allergy Organ J. 2018;73(8):1575‐1596. 10.1111/all.13384 29318628

[18] Busse PJ , Christiansen SC , Riedl MA , et al. US HAEA Medical Advisory Board 2020 guidelines for the management of hereditary angioedema. J Allergy Clin Immunol Pract. 2021;9(1):132‐150.e3. 32898710 10.1016/j.jaip.2020.08.046

[19] Nicola S , Rolla G , Brussino L . Breakthroughs in hereditary angioedema management: a systematic review of approved drugs and those under research. Drugs Context. 2019;8:212605. 31645881 10.7573/dic.212605 PMC6788388

[20] Bork K , Bygum A , Hardt J . Benefits and risks of danazol in hereditary angioedema: a long‐term survey of 118 patients. Ann Allergy Asthma Immunol. 2008;100(2):153‐161. 18320917 10.1016/S1081-1206(10)60424-3

[21] Craig T , Busse P , Gower RG , et al. Long‐term prophylaxis therapy in patients with hereditary angioedema with C1 inhibitor deficiency. Ann Allergy Asthma Immunol. 2018;121(6):673‐679. 30056152 10.1016/j.anai.2018.07.025

[22] Zuraw BL , Davis DK , Castaldo AJ , Christiansen SC . Tolerability and effectiveness of 17‐alpha‐alkylated androgen therapy for hereditary angioedema: a re‐examination. J Allergy Clin Immunol Pract. 2016;4(5):948‐955.e15. 27329469 10.1016/j.jaip.2016.03.024

[23] Longhurst H , Cicardi M , Craig T , et al. Prevention of hereditary angioedema attacks with a subcutaneous C1 inhibitor. N Engl J Med. 2017;376(12):1131‐1140. 28328347 10.1056/NEJMoa1613627

[24] Zuraw BL , Busse PJ , White M , et al. Nanofiltered C1 inhibitor concentrate for treatment of hereditary angioedema. N Engl J Med. 2010;363(6):513‐522. 20818886 10.1056/NEJMoa0805538

[25] Banerji A , Riedl MA , Bernstein JA , et al. Effect of lanadelumab compared with placebo on prevention of hereditary angioedema attacks: a randomized clinical trial. J Am Med Assoc. 2018;320(20):2108‐2121. 10.1001/jama.2018.16773 PMC6583584 30480729

[26] Zuraw B , Lumry WR , Johnston DT , et al. Oral once‐daily berotralstat for the prevention of hereditary angioedema attacks: a randomized, double‐blind, placebo‐controlled phase 3 trial. J Allergy Clin Immunol. 2020 Oct 21. 10.1016/j.jaci.2020.10.015. Online ahead of print. 33098856

[27] Riedl MA , Banerji A , Manning ME , et al. Treatment patterns and healthcare resource utilization among patients with hereditary angioedema in the United States. Orphanet J Rare Dis. 2018;13(1):180. 30314518 10.1186/s13023-018-0922-3 PMC6186115

[28] US Food and Drug Administration (FDA) . Center for Biologics Evaluation and Research (CBER). The voice of the patient: hereditary angioedema. May 2018. https://www.fda.gov/files/about%20fda/published/The‐Voice‐of‐the‐Patient‐‐‐Hereditary‐Angioedema.pdf. Accessed January 28, 2021.

[29] Orladeyo . [package insert]. Durham, NC: BioCryst Pharmaceuticals, Inc.; 2020.

[30] Ohsawa I , Honda D , Suzuki Y , et al. Oral berotralstat for the prophylaxis of hereditary angioedema attacks in patients in Japan: a phase 3 randomized trial. Allergy. 2020 Nov 28. 10.1111/all.14670. Online ahead of print. PMC8247297 33247955

[31] National Institute of Allergy and Infectious Diseases . Division of microbiology and infectious diseases (DMID) adult toxicity table. November 2007 Draft. https://www.niaid.nih.gov/sites/default/files/dmidadulttox.pdf. Accessed July 6, 2020.

[32] Weller K , Groffik A , Magerl M , et al. Development and construct validation of the angioedema quality of life questionnaire. Allergy. 2012;67(10):1289‐1298. 22913638 10.1111/all.12007

[33] Weller K , Magerl M , Peveling‐Oberhag A , Martus P , Staubach P , Maurer M . The Angioedema Quality of Life Questionnaire (AE‐QoL) ‐ assessment of sensitivity to change and minimal clinically important difference. Allergy. 2016;71(8):1203‐1209. 27038109 10.1111/all.12900

[34] Atkinson MJ , Sinha A , Hass SL , et al. Validation of a general measure of treatment satisfaction, the Treatment Satisfaction Questionnaire for Medication (TSQM), using a national panel study of chronic disease. Health Qual Life Outcome. 2004;2:12. 10.1186/1477-7525-2-12 PMC398419 14987333

[35] Riedl MA . Critical appraisal of androgen use in hereditary angioedema: a systematic review. Ann Allergy Asthma Immunol. 2015;114(4):281‐288.e7. 25707325 10.1016/j.anai.2015.01.003

[36] Sheffer AL , Fearon DT , Austen KF . Hereditary angioedema: a decade of management with stanozolol. J Allergy Clin Immunol. 1987;80(6):855‐860. Erratum in: J Allergy Clin Immunol. 1988;81(6):1208. 3693762 10.1016/s0091-6749(87)80277-4

[37] Johnston DT , Henry Li H , Craig TJ , et al. Androgen use in hereditary angioedema: a critical appraisal and approaches to transitioning from androgens to other therapies. Allergy Asthma Proc. 2021;42(1):22‐29. 33349293 10.2500/aap.2021.42.200106

