# Supplementary material for: Long‐term safety and effectiveness of berotralstat for hereditary angioedema: The open‐label APeX‐S study
Source: Clin Transl Allergy. 2021 Jun 18;11(4):e12035. doi: 10.1002/clt2.12035 (PMC8221587; doi:10.1002/clt2.12035)
Supplement: Supplementary file 1 — Supplementary Material [file CLT2-11-e12035-s001.docx]

**SUPPORTING INFORMATION**

**Long-Term Safety and Effectiveness of Berotralstat for Hereditary Angioedema:**

**The Open-Label APeX-S Study**

Henriette Farkas, MD, PhD, DSc^1^; Marcin Stobiecki, MD, PhD^2^; Jonny Peter, MBChB, PhD^3,4^; Tamar Kinaciyan, MD^5^; Marcus Maurer, MD^6^; Emel Aygören-Pürsün, MD^7^; Sorena Kiani-Alikhan, MB, PhD, FRCP, FRCPath^8^; Adrian Wu, MD^9^; Avner Reshef, MD^10^; Anette Bygum, MD, DMSci^11-13^; Olivier Fain, MD^14^; David Hagin, MD, PhD^15^; Aarnoud Huissoon, MB, PhD^16^;
Miloš Jeseňák, MD, PhD, MBA, MHA^17^; Karen Lindsay, MB BCh, MRCP^18^; Vesna Grivcheva Panovska MD, PhD^19^; Urs C. Steiner, MD^20^; Celia Zubrinich, MB, BS, FRACP^21^;
Jessica M. Best, DHSc^22^; Melanie Cornpropst, PharmD, PhD^22^; Daniel Dix, BS^22^;
Sylvia M. Dobo, MD^22^; Heather A. Iocca, PhD^22^; Bhavisha Desai, PharmD^22^;
Sharon C. Murray, PhD^22^; Eniko Nagy, MD^22^; William P. Sheridan, MB, BS^22^

^1^Hungarian Angioedema Center of Reference and Excellence, Department of Internal Medicine and Hematology, Semmelweis University, Budapest, Hungary; ^2^Department of Clinical and Environmental Allergology, Jagiellonian University Medical College, Krakow, Poland; ^3^Allergy and Immunology Unit, University of Cape Town Lung Institute, Cape Town, South Africa; ^4^Division of Allergy and Clinical Immunology, Department of Medicine, University of Cape Town, South Africa; ^5^Medical University of Vienna, Department of Dermatology, Vienna, Austria; ^6^Dermatological Allergology, Department of Dermatology and Allergy, Charité - Universitätsmedizin Berlin, Berlin, Germany; ^7^Department for Children and Adolescents, University Hospital Frankfurt, Goethe University Frankfurt, Germany; ^8^Department of Immunology, Barts Health NHS Trust, Royal London Hospital, London, United Kingdom; ^9^Center for Allergy and Asthma Care, Central, Hong Kong, China; ^10^Angioderma Center, Barzilai University Medical Center, Ashkelon, Israel; ^11^Department of Dermatology and Allergy Centre, Odense University Hospital, Odense, Denmark; ^12^Department of Clinical Genetics, Odense University Hospital, Odense, Denmark; ^13^Clinical Institute, University of Southern Denmark, Odense, Denmark; ^14^Sorbonne Université, Service de Médecine Interne, AP-HP, Hôpital Saint-Antoine, Paris, France; ^15^Allergy and Clinical Immunology Unit, Department of Medicine, Tel Aviv Sourasky Medical Center and Sackler Faculty of Medicine, University of Tel Aviv, Tel Aviv, Israel; ^16^Department of Immunology, Birmingham Heartlands Hospital, University Hospitals Birmingham, United Kingdom; ^17^National Center for Hereditary Angioedema, Department of Pediatrics, Department of Pulmonology and Allergology, Comenius University in Bratislava, Jessenius Faculty of Medicine, Martin, Slovakia; ^18^Auckland DHB Clinical Immunology and Allergy, Auckland, New Zealand; ^19^University Clinic of Dermatology; Ss Cyril and Methodius University, Skopje, Macedonia; ^20^Department of Immunology, University Hospital Zurich; Zurich, Switzerland; ^21^Allergy, Asthma, and Clinical Immunology, Alfred Health, Melbourne, Victoria, Australia; ^22^BioCryst Pharmaceuticals, Durham, NC, USA

| **Table S1.** APeX-S Countries and Investigators | |
| --- | --- |
| **Country** | **Investigators** |
| Austria | Tamar Kinaciyan  Werner Aberer |
| Australia | Celia Zubrinich  William Smith  Jo Douglass  Michael O’Sullivan  Constance Katelaris  Stephen Adelstein  Michaela Lucas |
| Belgium | Rik Schrijvers |
| Denmark | Anette Bygum |
| France | Olivier Fain  Laurence Bouillet  David Launay |
| Germany | Marcus Maurer  Emel Aygören-Pürsün  Susanne Trainotti  Jens Greve |
| Hong Kong | Adrian Wu |
| Hungary | Henriette Farkas |
| Italy | Marco Cicardi  Andrea Zanichelli  Mauro Cancian  Massimo Triggiani |
| Israel | Avner Reshef  Aharon Kessel  David Hagin  Nancy Agmon-Levin |
| Macedonia | Vesna Grivcheva-Panovska |
| Netherlands | Danny Cohn |
| New Zealand | Karen Lindsay  Russell Barker |
| Poland | Marcin Stobiecki |
| Serbia | Saldana Andrejevic  Dusanka Markovic |
| Slovakia | Milos Jeseňák |
| South Africa | Jonny Peter |
| South Korea | Hye-Ryun Kang  Jung-Won Park  Sujeong Kim  Won Seon Koh  Young-Min Ye |
| Spain | Ramon Lleonart-Bellfill  Maria Baeza |
| Switzerland | Urs Steiner |
| United Kingdom | Sorena Kiani  Aarnoud Huissoon  Ania Manson  Saul Faust  Will Rae  Claire Bethune Mark Gompels |
| United States | H. Henry Li |
| Some centers had more than one principal investigator during the conduct of the study. | |

| **Table S2.** Overall Summary of AEs per 100 PYE With Medical Concepts | | | |
| --- | --- | --- | --- |
| **AEs per 100 PYE** | **Berotralstat**  **110 mg**  **(n=100)** | **Berotralstat**  **150 mg**  **(n=127)** | **Total  (N=227)** |
| Any event | 686 | 598 | 632 |
| Most common AEs (>15 per 100 PYE in any treatment group)^†,‡^ | | | |
| Upper respiratory tract infection | 87 | 92 | 90 |
| Abdominal pain | 78 | 40 | 55 |
| Headache | 55 | 28 | 39 |
| Diarrhea | 25 | 21 | 22 |
| Alanine aminotransferase increased | 9 | 17 | 14 |
| AE, adverse event; HAE, hereditary angioedema; MedDRA, Medical Dictionary for Regulatory Activities; PYE, person-year of exposure. ^†^Adverse events are coded using MedDRA version 19.1. The terms “abdominal pain,” “diarrhea,” and "upper respiratory tract infection” are medical concepts containing multiple preferred terms. “Abdominal pain” contains the preferred terms “abdominal pain,” “abdominal discomfort,” “abdominal pain upper,” “abdominal pain lower,” and “epigastric discomfort.” “Diarrhea” contains the preferred terms “diarrhea” and “feces soft.” “Upper respiratory tract infection” contains the preferred terms “nasopharyngitis,” “upper respiratory tract infection,” “viral upper respiratory tract infection,” “respiratory tract infection,” and “respiratory tract infection viral.” ^‡^HAE attacks were listed as an AE with >15 PYE in the 110-mg treatment group but were excluded from the table because HAE attacks were considered SAEs in certain regions based on their hospitalization practices. | | | |

**SUPPORTING METHODS**

**Inclusion criteria (Protocol version 4.0)**

1) Males and nonpregnant, nonlactating females aged ≥18 years (main study) or ≥12 to 17 years of age (substudy).

2) Patients with HAE type 1 or type 2 HAE who either have:

a) Participated in a berotralstat study, OR

b) In the opinion of the investigator, are expected to benefit from being treated with an oral treatment for the prevention of angioedema attacks and have a clinical diagnosis of HAE type 1 or type 2, defined as having a C1-INH functional level below 50% and a C4 level below the lower limit of the normal (LLN) reference range, as assessed during the screening period. In the absence of a low C4 value drawn during the intercritical period (ie, when patient is not having an HAE attack), one of the following is acceptable to confirm the diagnosis of HAE: 1) a *SERPING1* gene mutation known or likely to be associated with HAE type 1 or 2 assessed during the screening period; 2) a confirmed family history of C1-INH deficiency; 3) C4 redrawn and retested during an attack with the results below the LLN reference range measured during the screening period. For patients with a C1-INH function level greater than or equal to 50% but less than the assay LLN, a *SERPING1* gene mutation known or likely to be associated with HAE Type I or II, as assessed during the screening period OR a repeat test showing C1-INH functional level below 50% will be considered acceptable for enrollment.

3) Patient weight ≥40 kg.

4) Access to appropriate medication for the treatment of acute HAE attacks.

5) Female patients must meet at least one of the following requirements:

a) Be a woman of childbearing potential (defined as a nonmenopausal adult or adolescent female who has not had a hysterectomy, bilateral oophorectomy, or documented ovarian failure) who agrees to use at least an acceptable effective contraceptive method during the study and for a duration of 30 days after last dose of study drug. One or more of the following methods are acceptable:

- Surgical sterilization (ie, bilateral tubal occlusion or vasectomy of male partner)
- Placement of an intrauterine device (IUD) or intrauterine system (IUS) (implanted any time prior to or during screening)
- Progesterone-only (implantable or injectable only) hormonal contraception associated with inhibition of ovulation initiated at least 7 days prior to the screening visit
- Combined (estrogen- and progestogen-containing) oral, intravaginal, or transdermal hormonal contraception associated with inhibition of ovulation
- Male or female condom with or without spermicide
- Use of an occlusive cap (diaphragm, or cervical/vault caps) with spermicide (foam/gel/film/cream/suppository)

Female patients who report being postmenopausal for ≤2 years and have a follicle-stimulating hormone (FSH) level ≤40 mIU/mL must agree to use at least an acceptable effective contraceptive method and (as proposed above) during study and for 30 days after the last dose of study drug.

Female patients of childbearing potential who declare themselves as either sexually abstinent or exclusively having female sexual partners do not need to use an acceptable method of contraception. This declaration should be reviewed with the patient throughout the study to ensure continued accuracy. Abstinence in this study is defined as “true abstinence: when this is in line with the preferred and usual lifestyle of the patient.”

b) Be a woman of nonchildbearing potential (defined as postmenopausal for >2 years or having an FSH level >40 mIU/mL if postmenopausal ≤2 years or have had a hysterectomy, bilateral oophorectomy, or documented ovarian failure. Nonchildbearing potential may also be demonstrated in the previous study).

6) Male patients must comply with the following requirements through the end of the study:

a) Patients with female partners of childbearing potential (defined as postmenopausal ≤2 years or a nonmenopausal female who has not had a hysterectomy, bilateral oophorectomy, or documented ovarian failure) must agree to utilize at least 1 acceptably effective contraceptive method. At least 1 or more of the following methods are acceptable:

- Surgical sterilization (ie, vasectomy or bilateral tubal occlusion of a female partner)
- Placement of an IUD or IUS
- Any form of hormonal contraception (oral, implantable, injectable, intravaginal, or transdermal)
- Use of a condom with or without spermicidal foam/gel/film/cream/suppository
- Partner’s use of an occlusive cap (diaphragm, or cervical/vault caps) with spermicidal foam/gel/film/cream/suppository

b) Male patients who declare themselves as sexually abstinent are acceptable for the purposes of this study. Abstinence in this study is defined as “true abstinence: when this is in line with the preferred and usual lifestyle of the patient.”

7) Able to provide written, informed consent. Patients aged ≥12 to 17 years who are screened for the substudy must be able to read, understand, and be willing to sign an assent form in addition to a caregiver providing informed consent.

8) In the opinion of the investigator, the patient is able to adequately comply with all required study procedures for the duration of the study. The patient must demonstrate adequate compliance with all study procedures required, including diary recording of HAE attacks.

**Exclusion criteria (Protocol version 4.0)**

1) Pregnant, breastfeeding, or planned pregnancy during the study period.

2) Any clinically significant medical condition or medical history that, in the opinion of the investigator or sponsor, would interfere with the patient’s safety or ability to participate in the study.

3) Discontinuation of study drug due to hypersensitivity reaction to berotralstat in a prior study. This includes patients who had a rash of any severity identified as possibly, probably, or definitely related to active berotralstat in the previous study.

4) Dementia, altered mental status, or any psychiatric condition that would prohibit the understanding or rendering of informed consent or participation in the study.

5) Clinically significant abnormal ECG including, but not limited to, a QTcF >470 ms for women, a QTcF >450 ms for men, a PR interval >220 ms (both sexes), or ventricular and/or atrial premature contractions that are more frequent than occasional, and/or as couplets or higher in grouping.

6) Unacceptable noncompliance in a previous berotralstat study as assessed by the sponsor or investigator.

7) Any clinically significant history of angina, myocardial infarction, syncope, clinically significant cardiac arrhythmias, left ventricular hypertrophy, cardiomyopathy, or any other cardiovascular disease.

8) Known family history of sudden cardiac death. Family history of sudden death from HAE is not exclusionary.

9) History of or current implanted defibrillator or pacemaker.

10) Use of concomitant medications that are metabolized by CYP2D6, CYP2C9, CYP2C19, or CYP3A4 and that have a narrow therapeutic range, including those known to prolong the QT interval within 7 days of the baseline visit or planned initiation during the study.

11) Use of a medication that is transported by P-glycoprotein (P-gp) and has a narrow therapeutic range within 7 days of the baseline visit or planned initiation during the study.

12) Any laboratory parameter abnormality that, in the opinion of the Investigator, is clinically significant and relevant for this study.

13) Calculated creatinine clearance of ≤30 mL/min or AST or ALT value ≥3 times the upper limit of the normal (ULN) reference range value at screening or last available visit prior to enrollment.

14) Investigational drug exposure, other than berotralstat, within 30 days prior to the screening visit (or baseline if no screening visit).

15) Severe hypersensitivity to multiple medicinal products or severe hypersensitivity/anaphylaxis with unclear etiology.

16) History of alcohol or drug abuse within the previous year, or current evidence of substance dependence or abuse (self-reported alcoholic intake >3 units of alcohol/day).

17) For patients undergoing a screening visit, a positive screen for drugs of abuse (unless used as a medical treatment [eg, with a prescription]).

18) For patients undergoing a screening visit, current infection with hepatitis B virus (HBV), hepatitis C virus (HCV), or human immunodeficiency virus (HIV).

19) Patients with an immediate family relationship to either the sponsor’s employees, the investigator, or employees of the study site who are named on the delegation log.

20) Patients who are held in an institution by a government or judicial order.
